# A 3-year national DRL for CT in hybrid imaging study in Kuwait health environment—impact and implementation

**DOI:** 10.1093/bjro/tzae032

**Published:** 2024-10-04

**Authors:** Michael Masoomi, Latifah Al-Kandari, Iman Al-Shammeri, Hany Elrahman, Jehan Al-Shammeri

**Affiliations:** Department of Nuclear Medicine and Molecular Imaging, Adan Hospital, MOH, Hadiya, 46020, Kuwait; Research and Innovation Department, Portsmouth University Hospital, Portsmouth, United Kingdom; Department of Radiology Medical Imaging, Adan Hospital, MOH, Hadiya, 46020, Kuwait; Department of Nuclear Medicine and Molecular Imaging, Adan Hospital, MOH, Hadiya, 46020, Kuwait; Department of Nuclear Medicine and Molecular Imaging, Adan Hospital, MOH, Hadiya, 46020, Kuwait; Nuclear Medicine Department, Faculty of Medicine, Kuwait University, Safat, 13110, Kuwait

**Keywords:** PET-CT, NDRL, Nuclear Medicine, Kuwait

## Abstract

**Objective:**

Diagnostic reference levels (DRLs) for CT in PET-CT are limited, and published DRLs from other countries may not be directly applicable to the State of Kuwait (KW). The authors aimed to carry out the final phase of a 3-year study on DRLs in KW, supporting optimization and dose reduction as imaging technology advances.

**Methods:**

In this cohort study, 400 adult oncology patients from 8 PET-CT centres were included, following the same procedures as in the first (2018) and second (2020) years, in accordance with the MOH-KW Ethical Committee’s recommendations. The CT dose index (CTDIvol), dose-length product (DLP), and scan length were recorded, and the median, mean, standard deviation, as well as the 75th and 25th percentiles, along with the whole-body (WB) effective dose (ED), were calculated. Comparative studies were conducted to track implementation and identify any shortfalls.

**Results:**

In this study, half-body (HB) and WB scans accounted for 66% and 34% of the total 400 cases, respectively. The proposed local DRL practice among the 8 centres in the 2022 study exhibited a maximum variation of 25%, showing a 30% improvement over 2020. The achievable local DRL remained consistent with 2020 levels. Comparative results of the third quartile DLP (476 mGy cm) and CTDIvol (4 mGy) values for 2022 indicated lower values for the third phase (400 entries) compared to 2020, with a 1.5-fold variation in DLP. The calculated ED for WB scans ranged from 2.6 to 7.1 mSv, with mean values of 4.7 ± 1.25 mSv, using a conversion factor (*k* = 0.0093 mSv/mGy/cm). The 2022 proposed national diagnostic reference levels (NDRLs) for HB (469 mGy cm, 4.0 mGy) were lower than the Swiss National Data (620 mGy cm, 6.0 mGy) and France (628 mGy cm, 6.6 mGy), but slightly higher than those of the United Kingdom (400 mGy cm, 4.3 mGy), despite the Swiss having about 5000 entries, France 1000 entries, and the United Kingdom 370 HB entries.

**Conclusions:**

There was a 11.1% continuous improvement in NDRL for 2022 compared to 9.1% in 2020 and 13% in 2018, demonstrating a trend of enhanced optimization.

**Advances in knowledge:**

The data established a trend of NDRL for WBCT (PET-CT) that can serve as a national databank for ongoing optimization. This promotes improvements in patient protection and quality care within the clinical environment of the State of Kuwait, aligning with the strategic goals of Kuwait Vision-2035.

## Introduction

There is an increasing global focus on the careful management of radiation exposures from CT imaging, as CT examinations typically involve higher radiation doses compared to most other medical X-ray procedures. A widely accepted method for optimizing medical radiation exposures, recommended by the International Commission on Radiological Protection (ICRP)^1^^,^^2^ and the International Atomic Energy Agency (IAEA),^3^ is the establishment and use of national, regional, and local diagnostic reference levels (DRLs). The CT dose measurement concept is based on the CT dose index (CTDI), which represents the average absorbed dose of contiguous slices.^4–6^ DRLs for the volumetric CTDI are well-established for dedicated CT procedures on specific body regions. However, comparable reference levels for whole-body (WB) and half-body (HB) CT used in PET-CT examinations are limited.^7^ Reference levels in medical imaging serve various purposes, often with differing degrees of clinical and technical specificity depending on the objective. At least 3 general aims can be identified: (1) to improve regional, national, or local distributions for general medical imaging tasks by identifying and reducing the number of unjustified high or low values in the distribution; (2) to promote good practices for more specific medical imaging tasks; (3) to promote an optimum range of values for a specified medical imaging protocol.^8^

DRLs are not regulatory or punitive limits and can be exceeded when clinically necessary. However, they serve as thresholds where reasons for exceeding should be investigated. While DRLs offer an initial target for optimization, it is often possible to obtain clinically sufficient images at doses below these levels.^4^^,^^9^ The 2 most common dose indices for CT are the volume CT dose index (CTDIvol, measured in mGy) and dose-length product (DLP, measured in mGy·cm). These values are calculated in a standardized manner across all CT models. Since these indices represent the radiation output measured directly during the CT examination, they provide a direct means to compare examination protocols and offer potential for patient dose reduction.

The State of Kuwait, with a population of approximately 4 300 000 people, including 1 200 000 Kuwaiti nationals and the rest expatriates, provides secondary care through 6 general hospitals and several specialized units. At the time of this study, there were only 8 molecular imaging centres, all located in government hospitals. This multicentre collaborative research collected data from CT (PET-CT) hybrid imaging systems currently in use across the State of Kuwait hospitals to analyse results and establish an NDRL (national diagnostic reference level) baseline based on a 3-year study. Countries such as the United Kingdom, Switzerland, France, and several in the region (eg, Saudi Arabia, UAE, Jordan) have published their NDRL values in recent years.^6^^,^^10–14^ However, these values, which may involve different imaging practices and technology, may not be directly applicable to Kuwait's specific circumstances or the types of examinations and procedures covered by their NDRLs, especially when clinical indications differ.

This work details a specific data collection approach, utilizing the third quartile values of CTDIvol and DLP in accordance with the IPEM-UK framework^15^ and ICRP recommendations,^1^^,^^2^ to analyse CTDIvol and DLP values at sites conducting WB oncology PET-CT examinations. The aim was to set an NDRL and assess its impact on patients’ radiation dose in terms of effective dose (ED) in the State of Kuwait. The study was part of a comprehensive national survey conducted during the third year of a 3-year audit proposal. It received approval from the medical ethics committee of the Ministry of Health-Kuwait, and all participants provided informed written consent for the collection of personal and medical data.

## Methods

In our previous study in 2020,^16^ a maximum variation of up to two and a half times in LDRL and DLP among the 8 PET-CT centres in the State of Kuwait highlighted the necessity for ongoing assessment of national DRLs. Monitoring these trends aims to minimize radiation doses and their impact on patients. The current studies involved 8 PET-CT centres, aggregating 400 datasets compared to 197 and 309 datasets in 2018 and 2020, respectively.^16^^,^^17^ The focus encompassed all PET-CT imaging systems and procedures across various locations and system numbers within each centre. Data collection adhered to the ethical guidelines of the State of Kuwait, involving adult patients. All collected data were anonymized, and each centre was assigned a reference number to benchmark their doses against established NDRLs. The primary objective was to estimate typical patient dose levels under prevailing practices for adult patients, guided by the following considerations:

Inclusion criteria:

- All adult patients over 18 years of age, both Kuwaiti and non-Kuwaiti, who underwent a PET-CT scan during 2022.

Exclusion criteria:

- Paediatric patients, due to the limited availability of data.
- All topograms (scanograms) and monitoring steps used in contrast-enhanced CT acquisitions.

To minimize the influence of centres providing a disproportionately large number of entries, the data contribution from each centre was limited to a maximum of 50 entries—an increase of 20% over 2020 and 40% over 2018. To account for variations in dose recording, a systematic random sampling technique was employed, selecting every 10th individual patient to gather 50 adult patient data entries from each participating department over a 12-month period in 2022. A “data sheet” and “help sheet” for different scanner types were provided to facilitate the identification of parameter values on the scanners (Supplement materials S1 and S2). Displayed values of radiation dose quantities, including CTDIvol and DLP, for adult patients undergoing procedures for clinical indications were recorded.

To evaluate radiation doses from the CT component of the examination, DLP values extracted from scanner-generated dose reports were utilized along with a conversion factor (k) representing the normalized ED per DLP (mSv × mGy^−1^  × cm^−1^) specific to the scan region. For HB and WB scans, conversion values (k) of 0.015 mSv × mGy^−1^  × cm^−1^ and 0.0093 mSv × mGy^−1^  × cm^−1^, respectively, were applied,^18^^,^^19^ These coefficients were calculated as mean values for adult patient examinations across various CT scanner models operating at 120 kVp, incorporating tissue weighting factors from ICRP 103 and voxel phantoms from ICRP 110 (averaged for adult male and female).^20^^,^^21^ The ED, prerequisite for optimizing and monitoring radiation exposure in CT (PET-CT) examinations, was estimated individually for each centre. Patients in this study were uniformly scanned without categorization by age, sex, or weight due to the use of automatic exposure control (AEC) across all nuclear medicine (NM) PET-CT centres, except one, that accounted for patient size differences. In the third year of the study (2022), as in the 2018 and 2020 studies,^16^^,^^17^ an anthropomorphic phantom imaging was conducted using a local GE PET-CT 710 scanner (GE Healthcare (Milwaukee, Wisconsin, USA) to evaluate CTDIvol and DLP. These data served as a complementary to verify the reliability and accuracy of the adopted procedures in 2022 for comparative analysis. The acquired images were saved in DICOM format for subsequent processing and analysis.

Data were collected retrospectively and analysed using procedures consistent with those from the first and second years of the study^16^^,^^17^ to ensure close comparability for national comparisons among the clinical centres in the State of Kuwait. The authors focused on specific procedures to mitigate uncertainty in data collection and entry by implementing standardization, training, double data entry, descriptive analysis, and feedback loops throughout the study. Despite these measures, no significant effects were noted. Most of PET-CT imaging focused on oncology scans, with other types of imaging were scarce and being excluded. Several protocols used in Kuwait were similar to those in the United Kingdom, Switzerland, and France, allowing for direct comparison of DRLs established through national surveys conducted in those countries (47 PET-CT centres in the United Kingdom, 16 in Switzerland, and 56 in France) regarding PET-CT oncology procedures.^6^^,^^10^^,^^11^ This comparison aimed to determine whether our results exceeded or fell below the published DRLs of these countries. CTDIvol served as the primary metric for comparison, while DLP was influenced directly by the scan length (SL) used at individual centres.

### Statistical analyses

Statistical analysis of CTDIvol, DLP, and SL was conducted, considering the goals of attenuation correction and localization. To ensure accuracy, analyses were performed separately for each centre. The Rounded third quartile (75th percentile) and first quartile (25th percentile) values of CTDIvol and DLP were used to suggest NDRLs and achievable doses, respectively, as a further optimization aid. Descriptive statistics were calculated including median, mean, standard deviation, minimum, maximum values, and 75th and 25th percentiles of the combined data. Changes in CTDI_vol_ for PET/CT system across reporting years were examined using a 1-way repeated-measures analysis of variance (ANOVA). Statistical significance was determined by a *P* value of less than 0.05. While the fundamental concept of ED remains unchanged under new ICRP recommendations, important aspects of its calculation have been updated, resulting in changes in dose per unit exposure values since the previous UK CT survey of 2003. The Pearson correlation coefficient was used to determine the linear relationship between numerical values for the increase or decrease of NDRLs, and these were compared to previous results from 2018 and 2020. Box plots and SPSS statistical software (version 26.0), were employed to generate additional graphs and analyses.

## Results

Seven out of 8 departments utilized Discovery™ GE PET-CT systems with Optima™ 64-slice CT components, including 2 digital GE PET-CT systems. The remaining department used a Philips Gemini PET-CT system. The AEC settings showed considerable variability in minimum and maximum mA values (50-150 mA for HB and 167-285 mA for WB oncology PET-CT examinations). All centres uniformly employed a CT tube voltage of 120 kV for PET-CT examinations without deviation. In the 2022 study, HB ^18^F-FDG PET-CT oncology scans accounted for 68% of the total collected data, compared to 65% in 2020 and 53% in 2018. WB PET-CT examinations (head to toe) represented 32% of the data, down from 35% in 2020 and 47% in 2018. The maximum variation in the mean SL values between the 8 PET-CT departments was 36% for HB scans and 11.5% for WB scans. Table 1 provides a summary of dose and SL statistics for combined SLs (HB + WB). The third quartile CTDIvol and DLP values were employed to establish local diagnostic reference levels (LDRLs), while the first quartile values were used to propose achievable reference levels for each participating department. Table 2 shows a maximum variation of 1.60 and 1.40 times for LDRL in DLP and CTDIvol, respectively, across the 8 centres. Table 3 presents the calculated mean and median NDRL and achievable reference levels for HB, WB, and HB + WB oncology datasets (further tabulated data provided in Supplement material S3).

**Table 1.** Summary statistics for the distribution of the scanner volume CT dose index and dose-length product for the protocol list (WB + HB) of each centre using PET-CT.

Centre	Protocol application (AC and L)	CTDIvol (mGy)					DLP (mGy × cm)					Scan length (cm)				
		Median	Mean	SD	Min	Max	Median	Mean	SD	Min	Max	Median	Mean	SD	Min	Max
1	PET oncology (WB + HB): 50 N	4.0	4.0	1.5	2.0	9.0	4.3	3.0	441	492	196	110	1072	505	405	109
2	PET oncology (WB + HB): 50 N	4.0	4.2	1.2	2.0	9.0	5.0	4.0	489	508	105	328	980	547	441	109
3	PET oncology (WB + HB): 50 N	4.0	4.4	1.5	1.3	8.0	5.0	3.0	738	716	246	66	1307	855	549	168
4	PET oncology (WB + HB): 50 N	3.0	3.5	1.9	1.0	9.0	4.0	2.0	383	424	227	136	890	629	220	109
5	PET oncology (WB + HB): 50 N	4.0	4.0	1.7	2.0	9.0	4.0	3.3	450	505	154	211	1022	546	417	109
6	PET oncology (WB + HB): 50 N	3.0	3.9	1.8	2.0	10.0	5.0	2.3	364	466	263	72	1288	608	294	122
7	PET oncology (WB + HB): 50 N	3.0	3.4	2.2	1.1	11.6	3.6	2.2	343	405	267	37	1328	526	225	114
8	PET oncology (WB + HB): 50 N	3.0	3.7	1.1	2.0	6.0	4.8	3.0	471	486	162	200	921	544	393	114

Abbreviations: AC = attenuation correction; CTDIvol = CT dose volume; DLP = dose-length product; HB = half body; L = localization; N = number of entries; TB = total body; WB = whole body.

**Table 2.** Proposed and achievable LDRL for the suggested clinical NM examination protocol (WB + HB) at each centre using PET-CT.

Centre	Protocol application (AC and L)	Proposed LDRL (75th percentile)		Achievable DRL (25th percentile)	
		CTDIvol (mGy)	DLP (mGy × cm)	CTDIvol (mGy)	DLP (mGy × cm)
1	PET oncology (WB + HB): 50 N	4.3	505	3	405
2	PET oncology (WB + HB): 50 N	5	547	4	441
3	PET oncology (WB + HB): 50 N	5	855	3	549
4	PET oncology (WB + HB): 50 N	4	629	2	220
5	PET oncology (WB + HB): 50 N	4	546	3.3	417
6	PET oncology (WB + HB): 50 N	5	608	2.3	294
7	PET oncology (WB + HB): 50 N	3.6	526	2.2	225
8	PET oncology (WB + HB): 50 N	4.8	544	3.0	393

Abbreviations: AC = attenuation correction; CTDIvol = CT dose volume; DLP = dose-length product; DRL = dose reference level; HB = half body; L = localization; LDRL = local dose reference level; N = number of entries; TB = total body; WB = whole body.

**Table 3.** Proposed and achievable NDRL for the suggested clinical NM protocols using PET-CT (based on mean and median values).

Category	Protocol application (AC and L)	Proposed LDRL (75th percentile)				Achievable DRL (25th percentile)			
		CTDIvol (mGy)		DLP (mGy × cm)		CTDIvol (mGy)		DLP (mGy × cm)	
1	PET oncology (HB): 289 N	4.5m	4.0m*	490m	469m*	3.8m	3.0m*	394m	331m*
2	PET oncology (WB): 111 N	4.0m	4.0m*	694m	673m*	3.1m	3.0m*	544m	510m*
3	PET oncology (WB + HB): 400 N	4.1m	4.0m*	506m	476m*	3.7m	3.0m*	455m	378m*

Abbreviations: AC = attenuation correction; CTDIvol = CT dose volume; DLP = dose-length product; DRL = dose reference level; HB = half body; L = localization; LDRL = local dose reference level; m = mean; m* = median; N = number of entries; NDRL = national diagnostic reference level; NM = nuclear medicine; WB = whole body.

Table 4 presents the calculated third quartile values for DLP (469 mGy cm) and CTDIvol (4.0 mGy) for HB, WB, and combined HB + WB CT (PET-CT) scans conducted in the State of Kuwait. These values are compared with DLP and CTDIvol data from the United Kingdom, Switzerland, and France for 2022. For the combined HB + WB scans in 2022, the percentage deviations from the NDRL ranged from 0% to −25% across all departments. Additionally, the ED was calculated for HB, WB, and combined HB + WB scans using 2 different conversion factors. The HB ED ranged from 4.6 to 11.5 mSv (mean value: 7.6 ± 2.02 mSv), while the WB ED ranged from 2.8 to 7.1 mSv (mean value: 4.6 ± 1.25 mSv). Table 5 shows a very strong positive Pearson correlation (*r* = 0.984) between ED results in 2022 and 2020, and a positive correlation (*r* = 0.550) between ED results in 2022 and 2018.

**Table 4.** Comparison of proposed NDRL for AC and localization product for the suggested clinical NM protocols using PET-CT: (based on mean and median values).

Centres	Protocol application (AC and L)	PET oncology (HB)	PET oncology (WB)	PET oncology (WB + HB)
1	United Kingdom: proposed NDRL CTDIvol (mGy)	4.3m, 4.3m*	–	–
	DLP (mGy × cm)	400m, 400m*	–	–
2	SWISS: proposed NDRL CTDIvol (mGy)	6.0m, 6.0m*	5.0m, 5.0m*	–
	DLP (mGy × cm)	620m, 620m*	720m, 720m*	–
3	FRANCE: proposed NDRL CTDIvol (mGy)	6.6m, 6.6m*	7.7m, 7.7m*	–
	DLP (mGy × cm)	628m, 628m*	762m, 762m*	–
4	KW: proposed NDRL-2018 CTDIvol (mGy)	5.5m, 5.2m*	4.2m, 4.2m*	4.4m, 4.6m*
	DLP (mGy × cm)	557m, 566m*	677m, 665m*	616m, 638m*
5	KW: proposed NDRL-2020 CTDIvol (mGy)	5.0m, 4.8m*	4.1m, 3.6m*	4.7m, 4.5m*
	DLP (mGy × cm)	537m, 514m*	684m, 536m*	575m, 507m*
6	KW: proposed NDRL-2022 CTDIvol (mGy)	4.5m, 4.0m*	4.0m, 4.0m*	4.1m, 4.0m*
	DLP (mGy × cm)	490m, 469m*	694m, 673m*	506m, 476m*

Abbreviations: AC = attenuation correction; CTDIvol = CT dose volume; DLP = dose-length product; DRL = dose reference level; HB = half body; KW = Kuwait; L = localization; LDRL = local dose reference level; m = mean; m* = median; N = number of entries; NDRL = national diagnostic reference level; NM = nuclear medicine; WB = whole body.

**Table 5.** Comparison of CT effective dose as a result of AC and localization product for the suggested clinical NM protocol using PET-CT: using the recommended published conversion factors; *K* = 0.015 (mSv/mGy/cm) and *K* = 0.0093 (mSv/mGy/cm).

Centre	ED (mSv)			ED (mSv)			ED (mSv)		
	Scan length—half body (HB)			Scan length—whole body (WB)			(HB + WB)		
	*K* = 0.015 mSv/mGy/cm			*K* = 0.0093 mSv/mGy/cm			*K* = (0.015 + 0.0093) mSv/mGy/cm		
		Median	Mean		Median	Mean		Median	Mean
1	PET oncology (HB): 29 N	6.8	6.7	PET oncology (WB): 21 N	4.2	4.2	PET oncology (WB + HB): 50 N	5.5	5.4
2	PET oncology (HB): 41 N	7.7	7.7	PET oncology (WB): 9 N	4.8	4.7	PET oncology (WB + HB): 50 N	6.2	6.2
3	PET oncology (HB): 4 N	7.6	8.9	PET oncology (WB): 46 N	4.7	5.5	PET oncology (WB + HB): 50 N	6.1	7.2
4	PET oncology (HB): 40 N	10.7	11.5	PET oncology (WB): 10 N	6.6	7.1	PET oncology (WB + HB): 50 N	8.6	9.3
5	PET oncology (HB): 34 N	7.4	8.5	PET oncology (WB): 16 N	4.6	5.3	PET oncology (WB + HB): 50 N	6.0	6.9
6	PET oncology (HB): 41 N	5.1	5.3	PET oncology (WB): 9 N	3.2	3.3	PET oncology (WB + HB): 50 N	4.1	4.3
7	PET oncology (HB): 40 N	4.2	4.6	PET oncology (WB): 10 N	2.6	2.8	PET oncology (WB + HB): 50 N	3.4	3.7
8	PET oncology (HB): 36 N	7.4	7.4	PET oncology (WB): 14 N	4.6	4.6	PET oncology (WB + HB): 50 N	6.0	6.0

Abbreviations: AC = attenuation correction; ED = effective dose; HB = half body; K = conversion factor; N = number of entries; NM = nuclear medicine; WB = whole body.

**Figure 1.**
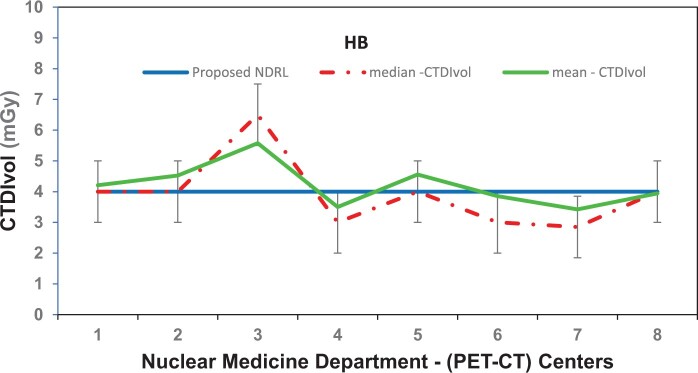
CTDIvol (75th percentile) for each PET-CT department, compared to the proposed NDRL for HB data. Abbreviations: CTDIvol = CT dose volume; HB = half body; NDRL = national diagnostic reference level.

**Figure 2.**
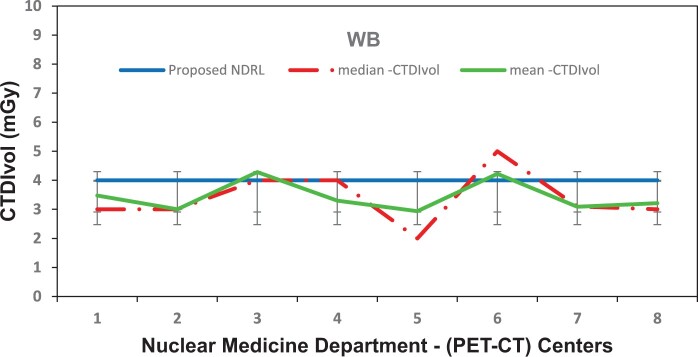
CTDIvol (75th percentile) for each PET-CT department, compared to the proposed NDRL for WB data. Abbreviations: CTDIvol = CT dose volume; NDRL = national diagnostic reference level; WB = whole body.

**Figure 3.**
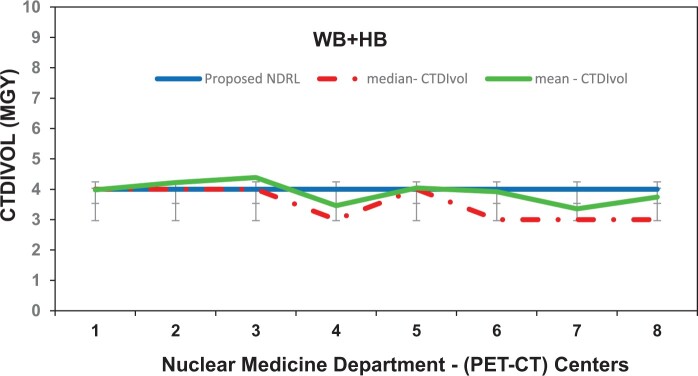
CTDIvol (75th percentile) for each PET-CT department, compared to the proposed DRL for WB + HB data. Abbreviations: CTDIvol = CT dose volume; DRL = diagnostic reference level; HB = half body; WB = whole body.

**Figure 4.**
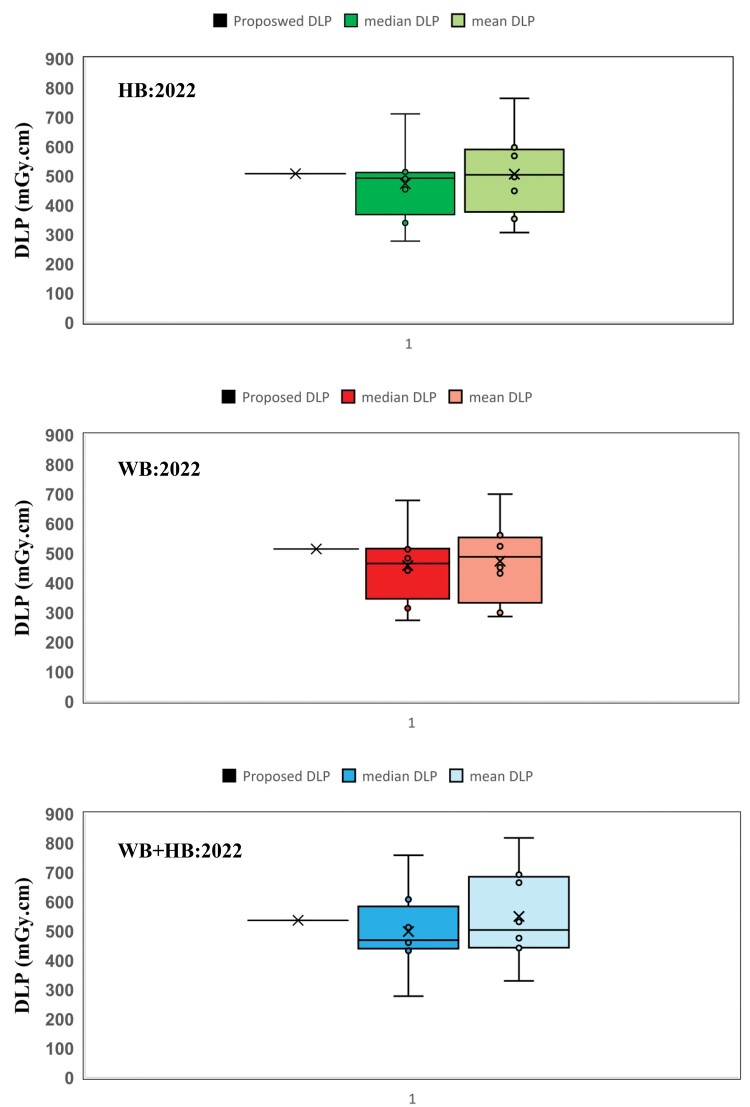
Box plots of DLP (median) based on HB, WB, and WB + HB data for each PET-CT department compared to the proposed related NDRL (75th percentile). Abbreviations: DLP = dose-length product; HB = half body; NDRL = national diagnostic reference level; WB = whole body.

**Figure 5.**
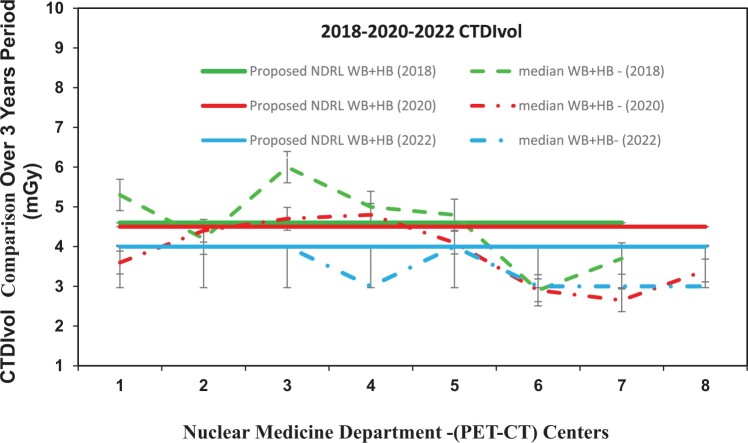
WB + HB CTDIvol (median) comparison with the proposed NDRL (75th percentile) at each PET-CT department in 2018, 2020, and 2022. Abbreviations: CTDIvol = CT dose volume; HB = half body; NDRL = national diagnostic reference level; WB = whole body.

**Figure 6.**
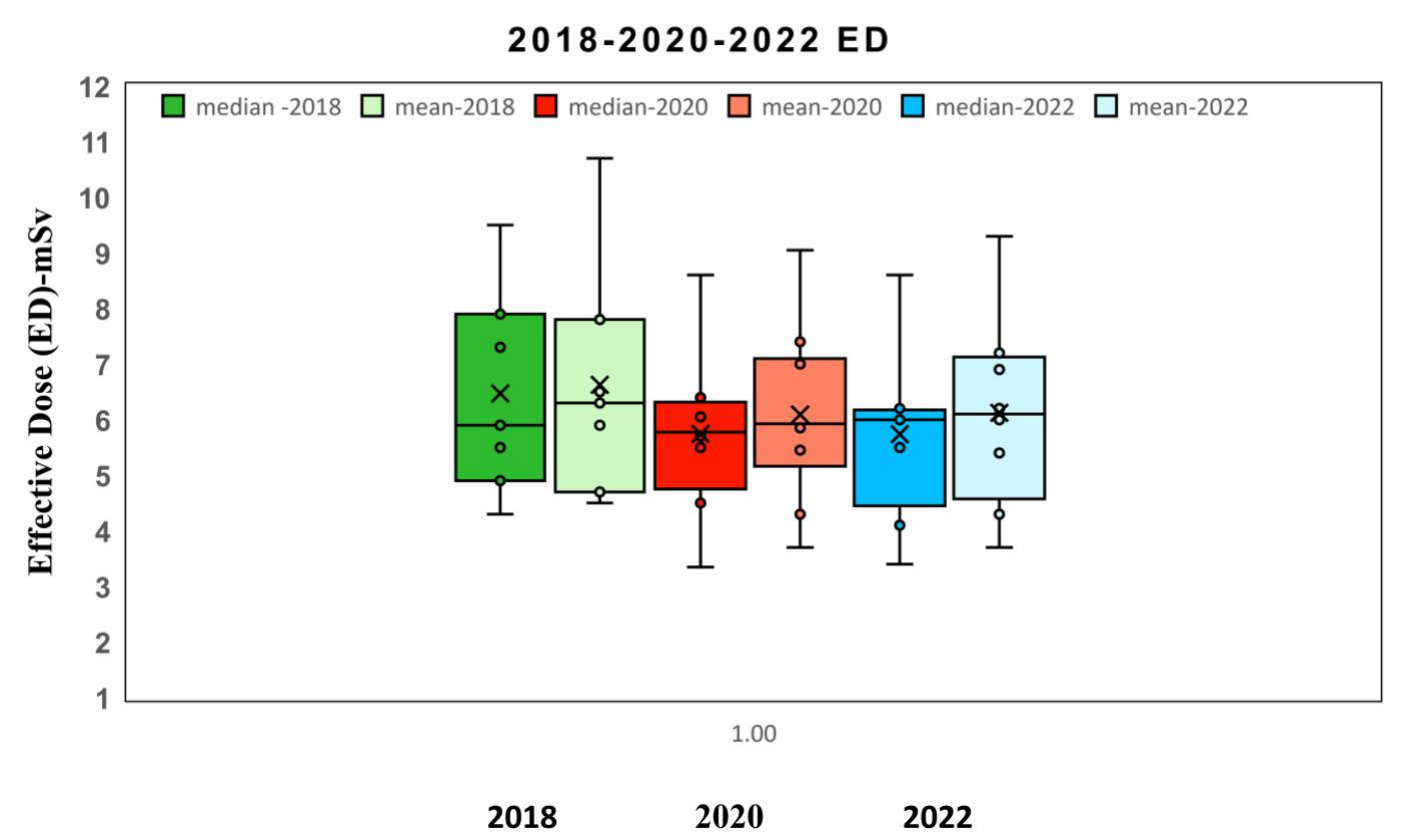
Box plots for comparison of CT ED for HB + WB data at each PET-CT department in 2018, 2020, and 2022. Abbreviations: ED: effective dose; HB = half body; WB = whole body.

**Figure 7.**
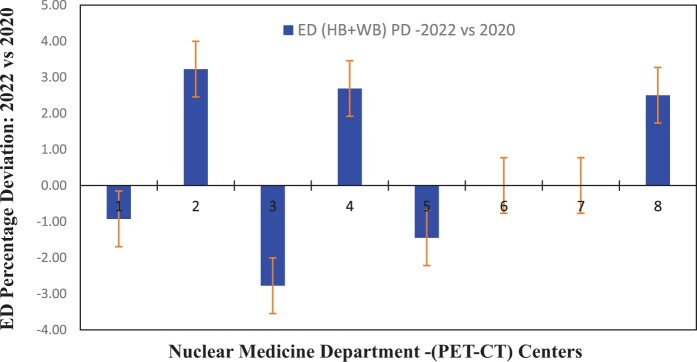
ED percentage deviation for 2022 compared to 2020 for HB + WB data. Abbreviations: ED: effective dose; HB = half body; WB = whole body.

Figures 1-3 depict the 75th percentile CTDIvol values for HB, WB, and HB + WB oncology examinations, used for attenuation correction and localization purposes across each department, compared against calculated NDRL. The results indicated considerable median (75th percentile) deviation in HB CTDIvol (64%) from the NDRL at 1 centre (no. 3), potentially due to the limited number of HB scans (no. 4) performed. Figure 4 illustrates box plots displaying the distribution of DLP-75th percentile data for HB, WB, and combined HB + WB scans, highlighting overall response patterns and variations relative to proposed DLP values for NM PET-CT departments. Figure 5 presents trends in CTDIvol variations from 2018 to 2022, comparing these against the national DRLs and providing a comparative analysis. The analysis helps in tracking the compliance and optimization efforts of each department over time. Changes in CTDIvol, analysed using a 1-way repeated-measures ANOVA, yielded significant differences with *P* = 0.368 for the comparison between 2022 and 2020, and *P* = 0.368 for the comparison between 2020 and 2018. The quantitative tabulated data has also been provided (Supplement material S4). Figure 6 provides box plot comparisons for HB + WB ED data at each PET-CT department over the years 2018, 2020, and 2022, illustrating overall response patterns and variations across the study period. Figure 7 shows the ED percentage deviation for 2022 compared to 2020, indicating continuous improvement in ED (HB + WB) in 2022 ranging from −2.78% to +3.23%. Using a 1-way repeated-measures ANOVA, the analysis yielded highly significant differences (*P* = 0.993) between the 2022 and 2020 EDs.

## Discussion

The implementation of NDRL is pivotal in standardizing radiation doses across medical imaging procedures, and so enhancing patient safety by minimizing unnecessary exposure. NDRLs serve as a benchmark for radiological practices, promoting the optimization of dose protocols to align with best practice standards and ultimately improve patient care outcomes. One of the notable strengths of this study is that it was the first to introduce NDRL for the CT component of PET-CT NM oncology examinations in the State of Kuwait. Spanning 3 biennial periods (2018, 2020, and 2022), this research provides a comprehensive assessment and observation of variations in dose product and application. This 3-year study addressed quantitative improvements to facilitate dose reduction in the use of WB/HB CT (PET-CT) across all participating government hospitals in the State of Kuwait. Additionally, it emphasizes efforts to reduce radiation doses, ensuring a comprehensive evaluation over an extended period. The following key points have been addressed:

(1) Avoided scan over-coverage by ensuring patients are positioned in the iso-centre of the machine and by considering the specific needs of obese patients. (2) Revised protocol parameters and addressed protocol variations. With the assistance of PET-CT provider GE Healthcare and individual department administrations, reviewed and updated all protocols on CT machines for each body region/clinical indication to initiate the necessary changes. (3) Optimized scan parameters to reduce radiation doses while maintaining acceptable image quality. (4) Utilized AEC to account for patient size differences and to aid in the optimization process. (5) Encouraged relevant staff to undergo further refresher training on the importance of radiation protection, proper patient positioning, and troubleshooting scan parameters to lower radiation dose levels.

This study has systematically gathered data from a representative cross-section of daily PET-CT practices in the State of Kuwait, primarily focusing on oncology examinations over a period of 3 years, conducted independently. Of the randomly selected patients, 41% were male and 59% were female, with a mean age of 57.7 ± 22.5 years for both genders. The mean body mass index for all patients was 29.8 ± 0.52 which falls within the overweight range (25.0-29.9).^22^ It is recommended that DRL values should be correlated with average patients’ weight 70 ± 10 kg. In this study mean patient weight was 77.5 ± 2 kg which appeared to be within the range.^23^ One of the primary objectives of this 3-year study was to monitor trends in variations with the aim of minimizing radiation doses and their impact on patients in the State of Kuwait. Except for 1 department, all utilized AEC, which adjusts tube current (mA) during scans based on patient thickness, tissue attenuation, length, and body asymmetry, offering significant dose reduction benefits. However, the minimum and maximum mA settings for AEC (50-150 mA for HB and 167-285 mA for WB PET-CT examinations) showed considerable variability, underscoring the need for ongoing evaluation and standardization. Nearly all PET-CT departments (except one) employed Adaptive Statistical Iterative Reconstruction (ASiR), a technique aimed at enhancing image quality while reducing radiation exposure. The mean and median SL values for HB scans were 106 cm and 109 cm, respectively, and for WB scans, they were 162 cm and 171 cm, respectively. These values were comparatively higher than those reported in the United Kingdom (95 cm, 94 cm for HB) and Switzerland (94 cm, 101 cm for HB; 119 cm, 128 cm for WB). The average lengths of males and females in the United Kingdom and Switzerland were not specified. In the 2022 study, HB ^18^F-FDG oncology scans accounted for 68% of the total, slightly higher than in 2018 (53%) and 2020 (65%).^16^^,^^17^ This increase reflects the implementation of revised protocols and optimized patient positioning, resulting in reduced patient radiation exposure. The range of CTDIvol doses for HB and WB scans across departments varied from 1.1 mGy to 11.6 mGy and 2 mGy to 8 mGy, respectively. The current data were compared with findings from the first and second years to observe trends and implemented optimization measures accordingly (Figure 5).

The NDRL for the 2022 study showed an 11% and 13% improvement over the results from 2020 and 2018, respectively, demonstrating a positive trend towards enhanced optimization and beneficial impact on clinical health practices in the State of Kuwait. The percentage deviation in ED for 2022 compared to 2020 ranged from −2.78% to +3.23%, indicating improvements in patient radiation exposure management across the participating departments for CT scans (HB + WB) in 2022. In comparison, as shown in Table 4, the third quartile DLP and CTDIvol values (469 mGy cm, 4.0 mGy) for the State of Kuwait were marginally higher than the UK NDRL (400 mGy cm, 4.3 mGy) but lower than those of the Swiss National NDRL (620 mGy cm, 6 mGy) and the French National NDRL (762 mGy cm, 7.7 mGy). Specifically, the Proposed NDRLs for WB scans were set at 694 mGy cm and 4.0 mGy, which were lower than those reported in Swiss national data (720 mGy cm, 5.0 mGy). The results from the State of Kuwait for the third year showed reasonable alignment with international standards, despite the smaller dataset comprising 265 entries for HB scans and 135 entries for WB scans. In comparison, Switzerland had approximately 5000 HB and 706 WB entries, the United Kingdom had 370 HB entries, and France had 1000 HB entries. The ICRP recommends using the third quartile of the distribution of individual median values as the DRL. However, this study presented both mean and median values for DRL to accommodate recommendations from various groups, including those from the United Kingdom, Switzerland, and France national surveys.

Several countries, such as the United Kingdom, Switzerland, France, Saudi Arabia, Jordan, the United Arab Emirates, and others^6^^,^^10–14^ have established practices and published NDRLs as part of their efforts to advance precision medicine and minimize radiation doses. However, these NDRL values, which may vary due to different imaging practices and technologies used, may not directly apply to the specific circumstances or types of examinations conducted in the State of Kuwait, particularly those with or without detailed clinical indications. Therefore, the proposed NDRLs for WB CT (PET-CT) established over 3 years could serve to support and encourage relevant bodies at the Ministry of Health—Kuwait to establish a data bank. This could serve as a monitoring tool to enhance the quality of care provided to the population of Kuwait. This research provides valuable insights into national survey data on CT dose metrics utilized in WB PET-CT imaging. For instance, the 75th percentile for CTDIvol averaged 4 mGy across all reporting years. These findings can serve as benchmark levels for the local and national institutions conducting oncologic WB PET-CT imaging and aid in optimizing their CT protocols. Appropriate local review and action should be taken when the value observed in practice is consistently outside the selected upper or lower level. This process helps avoid unnecessary tissue doses being received by patients in general and, therefore, helps avoid unnecessary risk for the associated radiation health effects. Additionally, the following steps could serve as effective strategies to achieve and maintain NDRLs and enhance dose reduction efforts:

(1) Appropriate local review and action when the value observed in practice is consistently outside the selected upper or lower level. (2) Regular and current quality assurance. (3) Reviewing and optimizing procedures for imaging equipment and specific clinical tasks and body dimensions. (4) Incorporating technological advancements, such as post-processing and iterative reconstruction in CT. (5) Conducting periodic training on imaging facilities and radiation protection.

The study should be revisited in the future to align with the continuous evolution of PET-CT technology and as part of the State of Kuwait’s ongoing healthcare optimization strategy. Establishing NDRLs and achievable dose targets is expected to standardize practices across the State of Kuwait and reduce discrepancies in future surveys, thereby promoting enhancements in patient safety and the delivery of quality care. The absence of image quality assessment in setting NDRLs remains a significant aspect that should be addressed in future studies.

## Conclusions

The DRLs proposed in this national study serve as a benchmark for departments to review and potentially optimize their imaging protocols and patient doses for CT used in WB PET-CT imaging. As the State of Kuwait embraces continuous healthcare modernization, periodic revisions of DRLs and optimization in line with technological advancements are essential. This 3-year study developed a set of CT (PET-CT) DRLs, with the 75th percentile for CTDIvol being 4 mGy in 2022 and averaging 4.37 mGy across all reporting years (2018, 2020, and 2022). The current NDRLs (2022) show a 11.1% improvement over 2020 and a 13.3% improvement over 2018, demonstrating the impact of DRLs on dose reduction and their implementation. The data produced can serve as a national reference for future assessments in the State of Kuwait, benefiting both Kuwaiti and non-Kuwaiti patients by enhancing healthcare services and focusing on dose reduction.

## Collaborating centres

- Adan Hospital—KW
- Farwaniya Hospital—KW
- Al-Jaber Hospital—KW
- Chest Diseases Hospital—KW
- Mubarak Hospital—KW
- Al-Jahra Hospital—KW
- Kuwait Cancer Control Center—KW
- Jaber Al Ahmed Molecular Imaging/KFAS—KW

## Supplementary Material

tzae032_Supplementary_Data
