# Supplementary material for: A 3-year national DRL for CT in hybrid imaging study in Kuwait health environment—impact and implementation
Source: BJR Open. 2024 Oct 4;6(1):tzae032. doi: 10.1093/bjro/tzae032 (PMC11495866; doi:10.1093/bjro/tzae032)
Supplement: tzae032_Supplementary_Data [file tzae032_supplementary_data.zip › SUP-1.pdf]

# SUPPLEMENT 1:

## NM Department - Hospital:

**Protocol (treatment region):**

### Scanner Information

| | |
|---|---|
| **Hospital Name*:** | |
| **Local system ID*:** | |
| **System manufacturer*:** | |
| **System model*:** | |

### Standard Protocol Settings

| | | |
|---|---|---|
| **Local protocol name*:** | | ** |
| **CTDI phantom size (cm) (i.e. 16 cm head or 32 cm body)*:** | | [a] |
| **Is Automatic Exposure Control (AEC) used?** | | [b] |
| **AEC name (e.g. AutomA, ZDOM, CARE Dose 4D, Sure Expose):** | | [c] |
| **AEC setting type (e.g. ref noise index, reference mAs, etc):** | | [d] |
| **AEC setting value (e.g. SD 7.5, ref mAs 200):** | | [e] |
| **minimum to maximum mA range for AEC (where applicable):** | | [f1] |
| **mA where AEC is not used** | | [f2] |
| **Is iterative reconstruction used?** | | |
| **Iterative recon type (e.g. ASIR, SAFIRE, iDose):** | | [g] |
| **Iterative recon value (e.g. ASIR 40%, SAFIRE 3, iDose level 4):** | | [h] |
| **Radiation beam collimation:- Beam width (mm):** | | [i] |
| **- Number of slices:** | | [j] |
| **- Detector size (mm) (e.g. 0.625,0.6):** | | [k] |
| **Tube voltage (kV):** | | [l] |
| **Tube rotation time (s):** | | [m] |
| **Primary image slice thickness (mm):** | | [n] |
| **Scan field of view (SFOV) (mm):** | | [o] |
| **Reconstruction field of view (DFOV) (mm):** | | [p] |
| **Axial or helical?** | | [q] |
| **Pitch (where applicable):** | | [r] |
| **Reconstruction algorithm or kernel (e.g. B30; FC17; Std)** | | [s] |
| **Is IV contrast used?** | | |
| **How many scan phases? e.g. contrast & non-contrast scans = 2 phases** | | |
| **Which other imaging modes are also used? (e.g. PET; MRI)** | | |

If a parameter value routinely varies, please indicate typical numerical range

**Calibration Data**

| | |
|---|---|
| Error of indicated $CTD_{vol}$ when last checked (+/- %) | |

* Denotes a mandatory field

** See notes on scanner specific help sheet

## Notes:

**Please include any other details and descriptions of your scan protocols e.g. non-contrast scan followed by contrast scan, 4D CT scan performed in two phases with 4D over tumour volume and 3D of whole lung, etc. Also include details of how other imaging modalities are used.**

## Your Information

| | |
|---|---|
| **Contact name*:** | |
| **Contact telephone no.*:** | |
| **Contact email *:** | |

***- Denotes a mandatory field**

**Local system ID*:** **Protocol No:**

| Patient No | Age at time of scan (yrs) | Body Mass (kg) | Scan length (mm) | Average effective mAs per rotation | CTDIvol (mGy)* | DLP (mGy.cm) (not including scouts)* | Total mAs (whole scan not including scouts) | Comments |
|---|---|---|---|---|---|---|---|---|
| 1 | | | | | | | | |
| 2 | | | | | | | | |
| 3 | | | | | | | | |
| 4 | | | | | | | | |
| 5 | | | | | | | | |
| 6 | | | | | | | | |
| 7 | | | | | | | | |
| 8 | | | | | | | | |
| 9 | | | | | | | | |
| 10 | | | | | | | | |
| 11 | | | | | | | | |
| 12 | | | | | | | | |
| 13 | | | | | | | | |
| 14 | | | | | | | | |
| 15 | | | | | | | | |
| 16 | | | | | | | | |
| 17 | | | | | | | | |
| 18 | | | | | | | | |
| 19 | | | | | | | | |
| 20 | | | | | | | | |
| 21 | | | | | | | | |
| 22 | | | | | | | | |
| 23 | | | | | | | | |
| 24 | | | | | | | | |
| 25 | | | | | | | | |
| 26 | | | | | | | | |
| 27 | | | | | | | | |
| 28 | | | | | | | | |
| 29 | | | | | | | | |
| 30 | | | | | | | | |

**Please complete for as many patients as possible up to a maximum of 30**

**Note:**
*** Denotes a mandatory field**

**Notes:**

1) **Total mAs delivered is not always shown on all scanners. Where it is available, it can usually be found on the patient scan record sheet. If not available just leave this column empty.**

2) **Please aim to provide DLP or CTDI data without the inclusion of dose from the scout views. If inclusion is unavoidable, please note that on this data sheet.**
