# Supplementary material for: A 3-year national DRL for CT in hybrid imaging study in Kuwait health environment—impact and implementation
Source: BJR Open. 2024 Oct 4;6(1):tzae032. doi: 10.1093/bjro/tzae032 (PMC11495866; doi:10.1093/bjro/tzae032)
Supplement: tzae032_Supplementary_Data [file tzae032_supplementary_data.zip › SUP-2.pdf]

GE scanners - help sheet

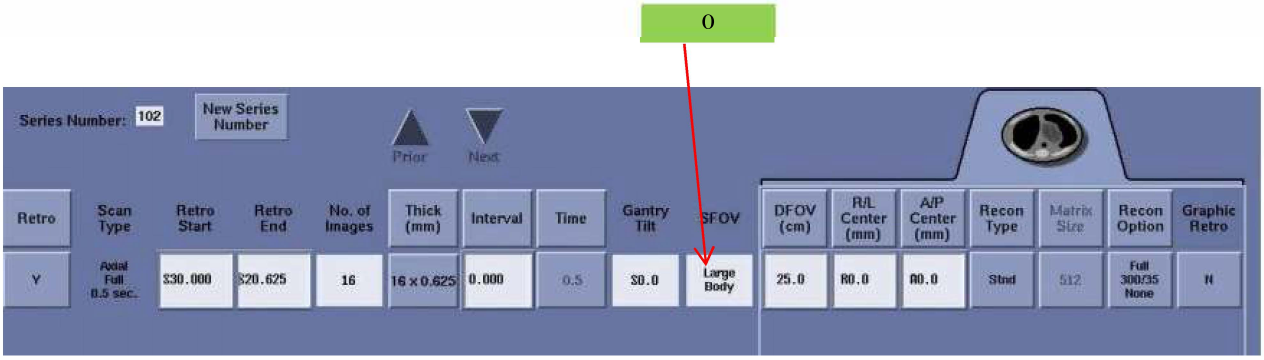

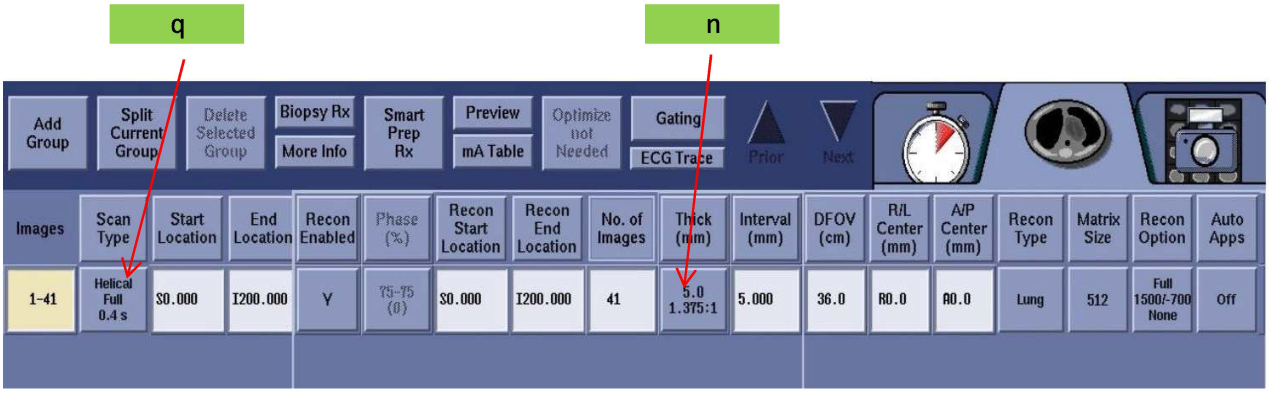

If auto mA

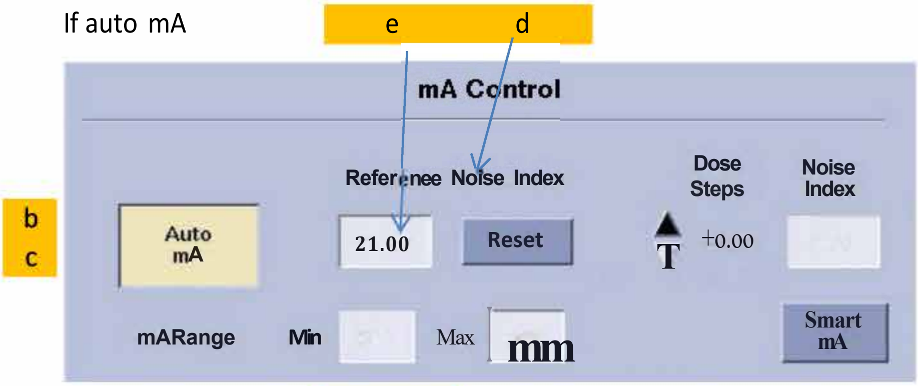

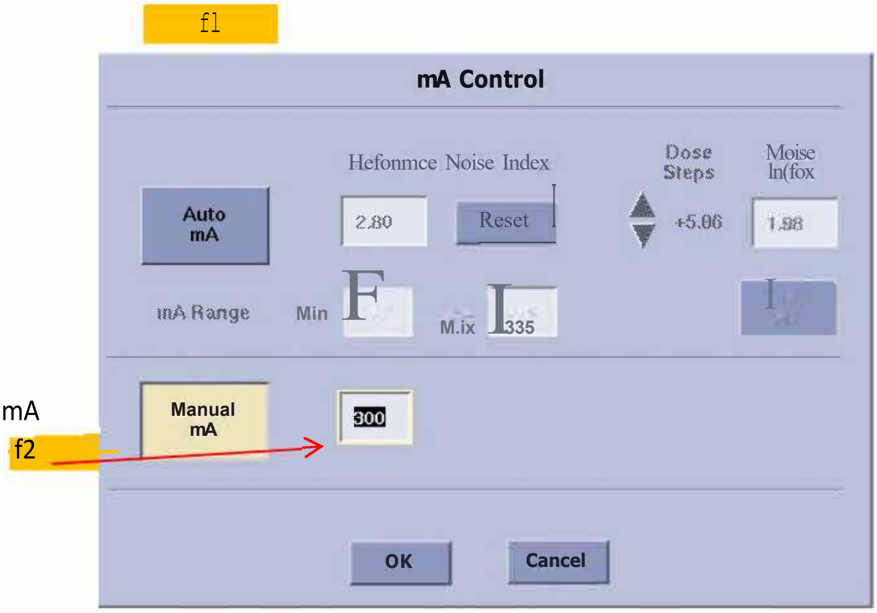

If manual mA

i

k

r

Pitch here is 0.984

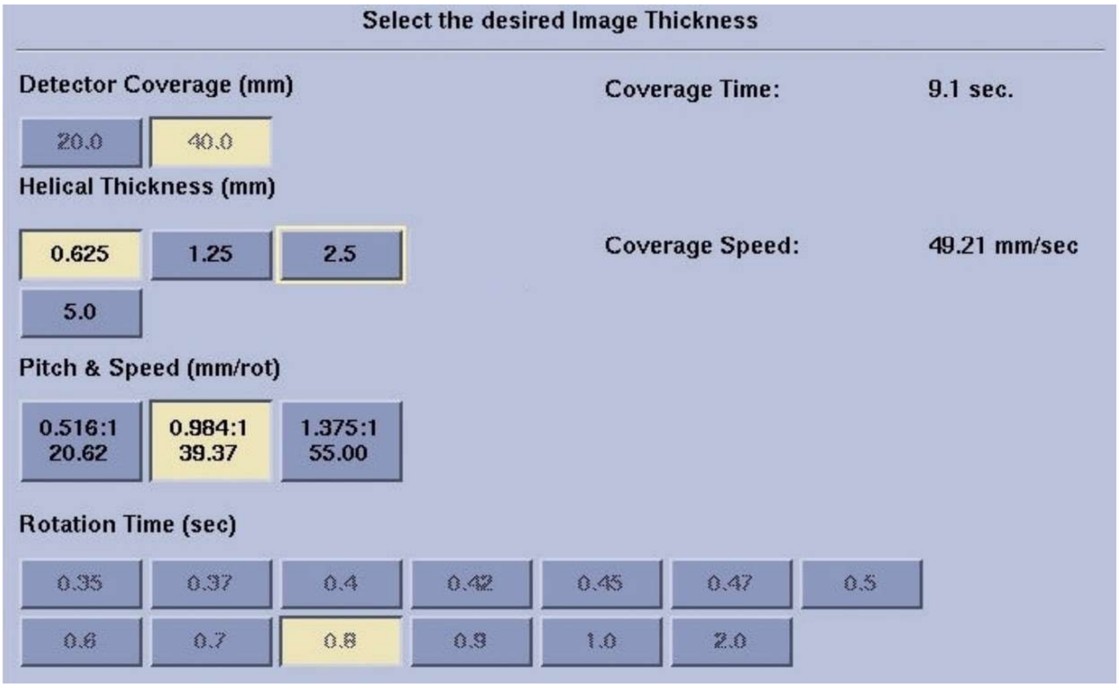

m

p

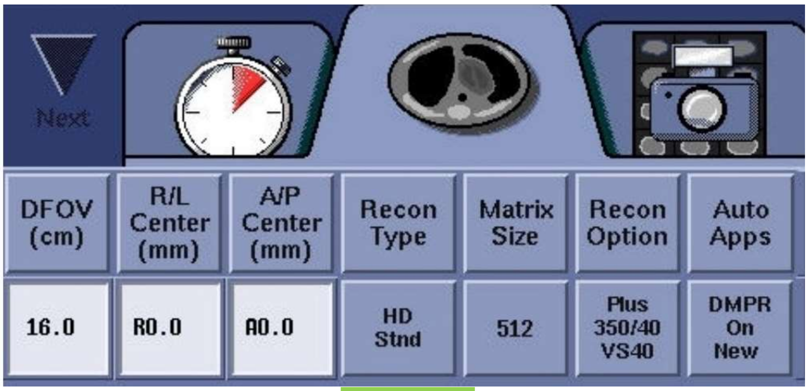

s

Example dose report showing delivered dose for, in this case, two axial scans

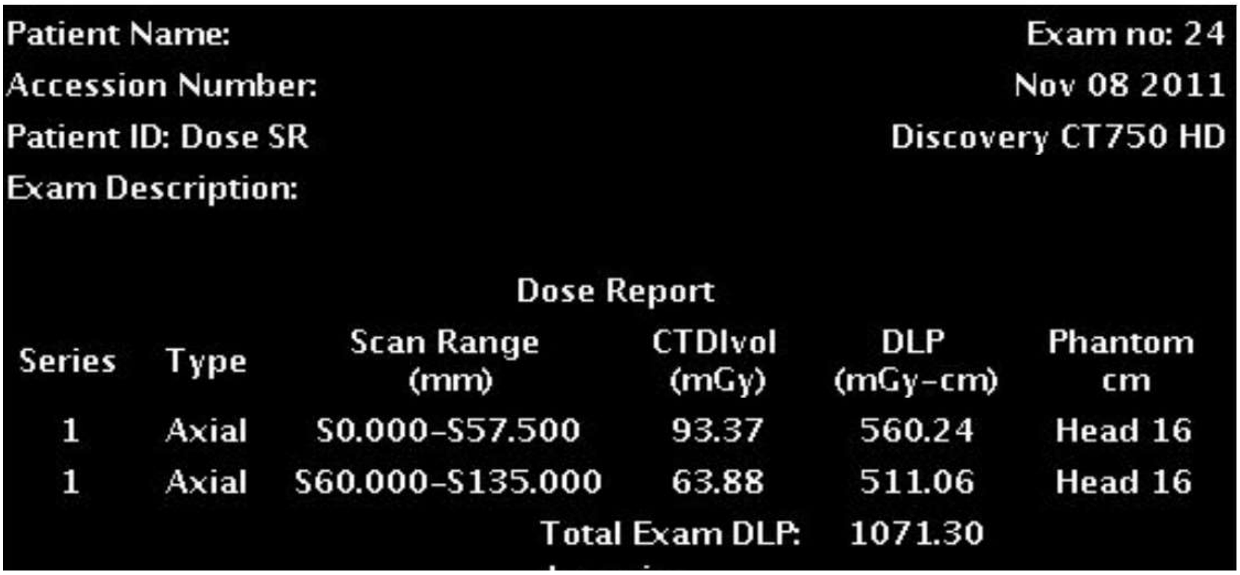

Patient Name: Exam no: 24

Accession Number: Nov 08 2011

Patient ID: Dose SR Discovery CT750 HD

Exam Description:

Dose Report

Series	Type	Scan Range (mm)	CTDIvol (mGy)	DLP (mGy-cm)	Phantom cm
1	Axial	S0.000-S57.500	93.37	560.24	Head 16
1	Axial	S60.000-S135.000	63.88	511.06	Head 16
		Total Exam DLP:		1071.30	

Total mAs if available & switched on will be shown on the dose report. (Not shown here)

If not available leave this part of the data sheet blank. a
