# Supplementary material for: A 3-year national DRL for CT in hybrid imaging study in Kuwait health environment—impact and implementation
Source: BJR Open. 2024 Oct 4;6(1):tzae032. doi: 10.1093/bjro/tzae032 (PMC11495866; doi:10.1093/bjro/tzae032)
Supplement: tzae032_Supplementary_Data [file tzae032_supplementary_data.zip › SUP-4.pdf]

SUPLLEMENT 4:

Proposed NDRL for the suggested clinical NM (WB+HB) examination at each center over the periods 2018, 2020 and 2022.

Hospital-NMC	2018 + 2020 +2022 RESULTS FOR COMPARISON								
	2018			2020			2022		
	Proposed NDRL	median	mean	Proposed NDRL	median	mean	Proposed NDRL	median	mean
	WB+HB 2018	WB+HB -CTDIvol 2018	WB+HB -CTDIvol 2018	WB+HB 2020	WB+HB -CTDIvol 2020	WB+HB -CTDIvol 2020	WB+HB 2022	WB+HB -CTDIvol 2022	WB+HB -CTDIvol 2022
1	4.6	5.3	4	4.5	3.6	3.6	4.0	4.0	4.0
2	4.6	4.2	2.4	4.5	4.4	4.4	4.0	4.0	4.2
3	4.6	6	5.5	4.5	4.7	5.0	4.0	4.0	4.4
4	4.6	5	4	4.5	4.8	5.3	4.0	3.0	3.5
5	4.6	4.8	3.4	4.5	4.1	4.6	4.0	4.0	4.0
6	4.6	2.9	3	4.5	2.9	2.6	4.0	3.0	3.9
7	4.6	3.7	2	4.5	2.7	2.7	4.0	3.0	3.4
8				4.5	3.4	3.8	4.0	3.0	3.7
