# Supplementary material for: A 3-year national DRL for CT in hybrid imaging study in Kuwait health environment—impact and implementation
Source: BJR Open. 2024 Oct 4;6(1):tzae032. doi: 10.1093/bjro/tzae032 (PMC11495866; doi:10.1093/bjro/tzae032)
Supplement: tzae032_Supplementary_Data [file tzae032_supplementary_data.zip › SUP-3.pdf]

SUPLLEMENT 3:

Source data for table 3:

"Proposed and achievable NDRL for the suggested clinical NM protocols using PET-CT: (Based on Mean &Median Value)."

PET-CT Centers	WB+HB				WB only				HB			
	mGy		mGy cm		mGy		mGy cm		mGy		mGy cm	
	CTDvol Median	CTDvol Mean	DLP Median	DLP -Mean	CTDvol Median	CTDvol Mean	DLP Median	DLP -Mean	CTDvol Median	CTDvol Mean	DLP Median	DLP -Mean
1	4.0	4.0	441	492	3.0	3.5	482	531	4.0	4	430	463
2	4.0	4.2	489	508	3.0	3.0	545	514	4.0	5	480	507
3	4.0	4.4	738	716	4.0	4.3	756	734	6.5	6	552	515
4	3.0	3.5	383	424	4.0	3.3	646	576	3.0	4	306	385
5	4.0	4.0	450	505	2.0	2.9	427	547	4.0	5	465	485
6	3.0	3.9	364	466	5.0	4.2	957	780	3.0	4	339	397
7	3.0	3.4	343	405	3.1	3.1	535	534	2.9	3	296	374
8	3.0	3.7	471	486	3.0	3.2	520	570	4.0	4	441	454
